# Bond strength between temporary 3D printable resin and conventional resin composite: influence of cleaning methods and air-abrasion parameters

**DOI:** 10.1007/s00784-022-04800-7

**Published:** 2022-11-28

**Authors:** Valerie Lankes, Marcel Reymus, Anja Liebermann, Bogna Stawarczyk

**Affiliations:** 1 Department of Prosthetic Dentistry, University Hospital, LMU Munich, Goethestraße 70, 80336 Munich, Germany; 2 Department of Conservative Dentistry and Periodontology, University Hospital, LMU Munich, Goethestraße 70, 80336 Munich, Germany; 3 Department of Prosthetic Dentistry, Faculty of Medicine and University Hospital Cologne, University of Cologne, Kerpener Str. 32, 50931 Cologne, Germany

**Keywords:** 3D resin, Cleaning, Air-abrasion, Surface properties, Bond strength, Fracture types

## Abstract

**Objectives:**

The influence of different cleaning methods, air-abrasion parameters, and aging on shear bond strength (SBS) and tensile bond strength (TBS) of 3D resin luted to composite resin.

**Materials and methods:**

Nine hundred resin substrates were 3D printed (D20II, Rapid Shape) and cleaned with either isopropanol (ISO), butyldiglycol-based solution (BUT), or centrifugation (CEN). After 24-h storage in 37 °C water, specimens were air-abraded (mean particle size 50 µm; *n* = 60) with either alumina at 0.1 MPa (AL0.1) or 0.4 MPa (AL0.4) and glass pearls at 0.1 MPa (GP0.1) and 0.4 MPa (GP0.4) or conditioned with visio.link (control) and luted with PanaviaV5. Initially (24 h, 37 °C water storage) or after aging (10,000 thermal cycles), SBS and TBS were measured, and fracture types were examined. Surface free energy (SFE) and roughness (Ra) were determined after air-abrasion. Kolmogorov–Smirnov, Kruskal–Wallis *H*, Mann–Whitney *U*, chi-square, and partial eta-squared were computed.

**Results:**

SBS measurements presented higher values than TBS (*p* < 0.001–0.033). Within the pretreatment groups, CEN showed the highest SBS and TBS values compared to cleaning with ISO or BUT (*p* < 0.001–0.040). Pretreatment with GP0.1 displayed the lowest bond strength values (*p* < 0.001–0.049), and mostly adhesive fractures occurred. The highest Ra values (*p* < 0.001) were observed for AL0.4 pretreatment.

**Conclusions:**

Pretreatment with AL0.4 and the control group mainly presented the highest bond strength values. Thermocycling had a positive effect on the bond strength.

**Clinical relevance:**

According to this study, 3D-printed restorations should be pretreated with AL0.4 or with visio.link before adhesive luting, regardless of their cleaning.

## Introduction 

The computer-aided design/computer-aided manufacturing (CAD/CAM) technology allows composite resin materials to be used for permanent indirect restorations. The term CAD/CAM stands for a variety of digitally supported techniques. For CAD/CAM polymers and composite resins, CAM is traditionally equivalent with the subtractive (milling) way of manufacturing [1]. Nowadays, additive manufacturing (AM), commonly known as 3D printing, is increasingly appreciated. Usually, the printing object is built up three-dimensional, layer by layer out of a vat of light-polymerizing resin by action of light, using stereolithography (SLA) or digital light processing (DLP) technology [2]. In contrast to milling and grinding, there is less restriction in object-geometry and waste of material. AM is already now well established in the prosthetic pretreatments such as bite splints, customized impression trays, surgical guides, and removable dental protheses. The latest material and printing research confirms that 3D-printed resin-based temporaries are suitable for long-term use [3]. Printed long-term temporaries present higher accuracy, better marginal fit [4], higher fracture resistance [5], and biocompatibility [6] compared to the conventionally manufactured ones. Up to now, only few 3D printable materials are available for fixed permanent restorations [7], but many manufacturers of 3D printable resins for long-term temporaries strive to obtain an approval under the medical device regulation, for the application as fixed dental protheses.

Besides mechanical and biological properties, a durable bond between restoration and the luting material is a crucial factor for a sufficient clinical long-term stability. The adhesion of composite material to tooth structure has already been extensively clarified and documented [8, 9]. In general, composite resin materials consist of a resin matrix of polymerized methacrylate, inorganic fillers, and (photo-)initiators. For milled composite resin restorations, the removed smear layer and unpolymerized carbon–carbon double bonds (free methacrylate) are important to create a strong adhesive bond by co-polymerizing the luting composite resin. The mechanical pretreatment of the bonding area is the most popular method for eliminating the smear layer, enlarging the surface area, and creating micro-mechanical retentions. Various air-abrasion powders with different mean sizes and pressures are described in literature [10–12]. Alumina powder displayed especially promising results [13] but has been criticized for damaging the surface, whereas air-abrasion with glass pearls would be sufficient [14]. Only a few in vitro studies have been concerned with the influence of air-particle abrasion on the surface properties of 3D printable resin restorations [15, 16]. None of them though takes air-abrasion pressure into account. However, with AM, there is no smear layer due to grinding or milling. Here, the post-processing procedures are important to be considered.

After the printing process, the objects must be freed from excess adherent uncured resin. Various cleaning methods are described in literature, whereby most resin manufacturers, despite lacking the scientific basis, suggest to simply rinse with isopropanol [17, 18]. This recommendation needs to be questioned, since solutions in particular may lead to changes in the surface structure of the printed object [18]. It is a necessity that the cleaned objects are being post-polymerized [19] by increasing converted carbon–carbon double bonds to stabilize mechanical and especially biological properties [19]. There is no literature yet, concerning the potential or limitations of post-processing procedures in combination with mechanical pretreatment with regard to the adhesion bond between the 3D resin and the luting composite resins. Therefore, the study at hand has been conducted.

The aim was to investigate the influence between three different cleaning methods and four different air-abrasion procedures, varying in pressure and air-abrasion agents, on the shear (SBS) and tensile (TBS) bond strength between 3D-printed temporary resin and a dual curing resin composite. The selected cleaning solution was either suggested by a manufacturer (isopropanyl alcohol) or specially developed for cleaning 3D-printed objects (InovaPrint wash). Additionally, centrifugation, as a physical cleaning method, was used since it is also recommended by some manufacturers. The centrifugal force has already been researched with regard to cleaning and mechanical properties of printed objects and has displayed promising results [18]. As a control group, a protocol with visio.link combined with 0.1 MPa alumina air-abrasion was chosen as this combination demonstrated good bond strength values (23.7–25.7 MPa) in various studies concerning the luting of CAD/CAM composite blocks [20, 21]. To investigate the bond strength of fixed dental prostheses, it is essential for in vitro studies to be as close to the clinic as possible; therefore, thermocycling as artificial aging was also included.

The null hypothesis stated that neither the cleaning method nor the pretreatment (air-abrasion powder and pressure) nor the aging regime nor the test method has an impact on the bond strength. Furthermore, the null hypothesis was that the air-abrasion shows no impact on the surface roughness and surface free energy.

## Material and methods

**Fig. 1:**
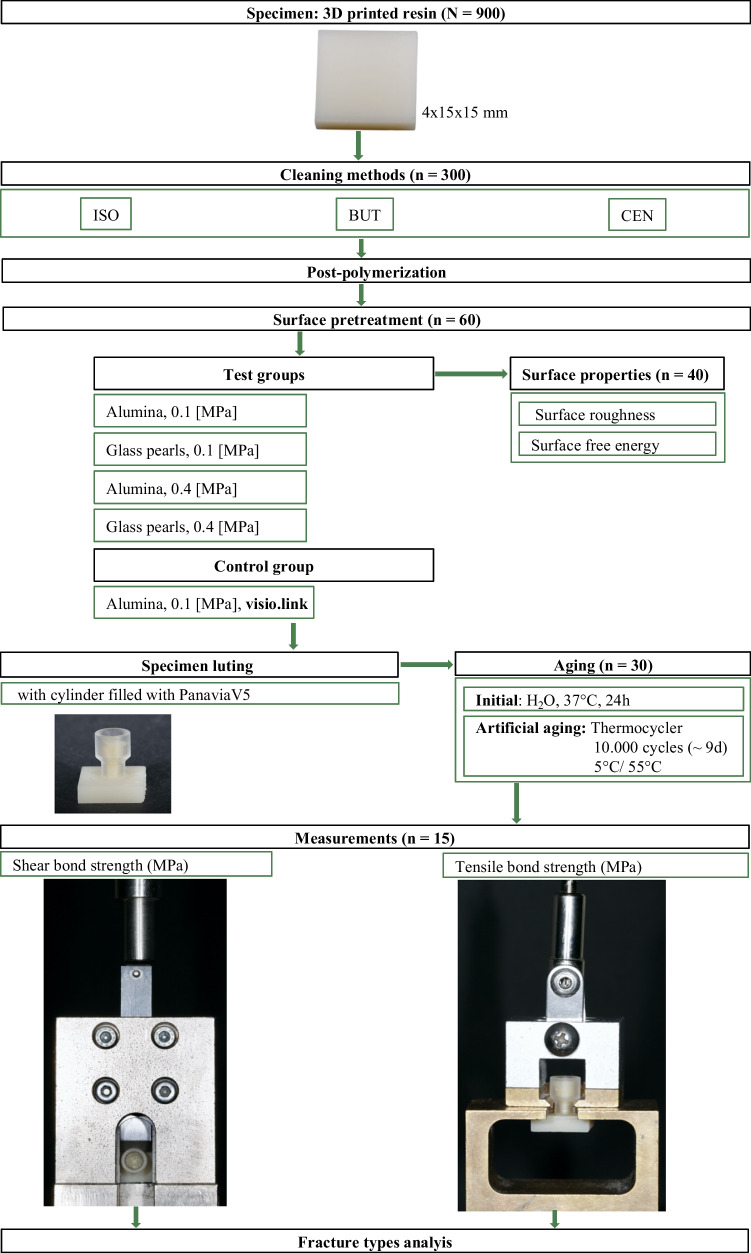
Study design

A specimen geometry (4 × 15 × mm) was digitally designed (Meshmixer software, Autodesk Inc., San Rafael, CA, USA) and exported as a STL file. A total of 900 resin specimens (printodent Generative Resin GR-17.1 temporary lt, Pro3dure medical GmbH, Iserlohn, Germany) were additively produced, vertically to the printer’s platform in a layer thickness of 50 µm by using the digital light processing (DLP) printer D20II (Rapidshape, Heimsheim, Germany) according to the manufacturer’s instructions. Before printing, the 3D resin was processed on a roller stirring device (LC-3D Mixer, NextDent, Soesterberg, Netherlands) for 30 min to achieve a sufficiently homogeneous distribution of the ingredients. An overview of the study design is presented in Fig. 1.

The printed specimens were divided into three groups (*n* = 300) and cleaned as follows:

1. Isopropanol (ISO) (100%, SAV LP GmbH, Flintsbach, Germany) for 4 min in an ultrasonic bath (Sonorex Super RK 102H, Bandelin, Berlin, Germany). The residue of the liquid was removed with compressed air.
2. Butyldiglycol-based cleaning solution (BUT) (InovaPrint Wash, hpdent GmbH, Gottmadigen, Germany) for 2 min in an ultrasonic bath as recommended by the manufacturer. The residue of the liquid was removed with compressed air.
3. Centrifugation (CEN) (Allegra X-15R, Beckman Coulter GmbH, Krefeld, Germany). Two specimens in each polypropylene conical tube (Cellstar Tubes 50 ml, Greiner Bio-One, Austria) were centrifugated with 600 G for 10 min.

All specimens were post-cured using OtoFlash G171 (NK-Optik, Baierbrunn, Germany) for 2000 flashes from each side (flashlight; wavelength range 280–700 nm, peaks at approximately 400 and 500 nm) and subsequently stored for 24 h in distilled water at 37 °C. The specimens were further divided into five subgroups (*n* = 60) and air-abraded with alumina or glass pearls with a mean particle size of 50 µm for 10 s (basis Quattro IS, Renfert, Hilzingen, Germany). The execution duration was controlled manually via a timer. The evaluated air-abrasion powders combined with specific pressures are listed in Table 1.

**Table 1:** Summary of pretreatment with abbreviations, material, manufacturers, composition, and lot numbers

Abbreviation	Pretreatment	Material	Manufacturers	Composition	Lot
AL0.1	Powder: alumina Pressure: 0.1 MPa	Strahlkorund	Orbis Dental Handelsgesellschaft mbH, Münster, Germany	Aluminia powder, mean particle size 50 µm	20,190,288
AL0.4	Powder: alumina Pressure: 0.4 MPa				
GP0.1	Powder: glass pearls Pressure: 0.1 MPa	Perlablast micro	Bego Bremer Goldschlägerei, Bremen, Germany	Lead-free sodium hydrogencarbonate glass pearls, mean particle size 50 µm	A46518
GP0.4	Powder: glass pearls Pressure: 0.4 MPa				

A blasting tool ensured 10 mm distance between the noodle and the specimen’s surface with an angle of 45°.

Then, all specimens were ultrasonically cleaned for 3 min in distilled water and carefully dried with compressed air.

As the control group, 180 specimens were, after air-abrasion with alumina at 0.1 MPa, additionally treated with visio.link (bredent, Senden, Germany). The conditioning agent was applied with a microbrush and then light cured for 90 s with a manufacturer-recommended light curing unit (bre.Lux Power unit, bredent) on the specimen’s surface. The pretreatment was performed immediately before bonding of the specimens.

An acrylic cylinder (SD Mechatronik, Feldkirchen-Westerham, Germany) with an inner diameter of 2.9 mm was positioned on each pretreated specimen’s surface, filled with a luting composite resin in shade A2 (Panavia V5, Kuraray Noritake, Okyama, Japan). Excess luting material around the cylinder on the luting area was carefully removed with a microbrush before polymerizing for 40 s (10 s from four different sides) using a LED light unit (Elipar Deep Cure-S, 3 M, Seefeld, Germany) with a wavelength of 430–480 nm and a light intensity of 1.480 mW/cm^2^. The cylinder was not disconnected before conducting the bond strength tests.

All specimens were subsequently stored in distilled water for 24 h at a temperature of 37 °C before half of the specimens were aged by a thermocycling process (Thermocycler, SD Mechatronik, Feldkirchen-Westerham, Germany). The artificial aging completed 10,000 thermal cycles between 5° and 55 °C remaining for 20 s in each bath.

### SBS and TBS measurements

SBS and TBS were carried out in a universal testing machine (Zwick 1445, Zwick, Ulm, Germany). For SBS, the compound surface was parallel to the loading direction, and the acrylic cylinder to the horizontal direction. The specimens were vertically loaded at a rate of 1 mm/min until fracture. For TBS, the specimens were fixed in a special holding device pulled apart by an upper chain with a crosshead speed of 5 mm/min until bond failed and calculated as follows: fracture load/bonding area (N/mm^2^ = MPa).

### Fracture types

The deboned area of each specimen was evaluated using a digital microscope magnification of 50 × (VHX-970F, Keyence, Osaka, Japan), and fractures were classified as follows:

i. Adhesive between the substrate and the luting composite
ii. Cohesive within the luting composite resin
iii. Cohesive within the 3D-printed resin
iv. Mixed cohesive

### SFE and Ra

From each of the four air-abrasion groups, 10 specimens were taken to conduct angle measurements (Easy Drop, Krüss, Hamburg, Germany) to determine SFE. Measurements were performed at room temperature by the sessile drop method with a defined volume of the test liquids which were distilled water and diiodomethane (CAS 75–11-6, Sigma-Aldrich, St. Louis, USA). Three drops of each liquid were generated on each specimen’s surface. After 5 s, a picture was taken, and the drop was analyzed with the tangent 1 method for distilled water or the circle method for diiodomethane by the used software (DSA 4, Drop Shape Analysis, Krüss). After specifying the baseline of the drop, the contact angle was calculated with the Owens–Wendt–Rabel–Kaelble method. Further, the same specimens were used for tactile Ra measurements by a profilometer (MarSur M 400, Mahr, Göttingen, Germany). Six measurements (3 × horizontal, 3 × vertical) were conducted on each specimen, with a length of 5.6-mm and 3-mm distance between the single tracks, determined Ra.

### Statistical analysis

The data were analyzed statistically with SPSS version 26.0 (IBM, SPSS, Statistics, Armonk, NY, USA). The normal distribution was analyzed using the Kolmogorov–Smirnov test. The global univariate ANOVA with partial eta-squared (*η*_P_^2^) were applied to figure out the impact of the tested parameters. The differences between the groups were analyzed non-parametrically with the Kruskal–Wallis *H* and multiple pairwise Mann–Whitney *U* test. For the correlation between SBS and TBS, the Spearman rho test was applied. The frequency of fracture types was analyzed by the chi-square test and Ciba-Geigy table. *p* values less than 0.05 were interpreted as statistically significant.

## Results

A deviation of the normal distribution was observed; therefore, the data were analyzed non-parametrically. Descriptive statistics with standard deviation (SD); 95% confidence intervals; and minimum, medium, and maximum are summarized in Tables 2 and 3. The highest impact on SBS and TBS was exerted by the test method (*η*_P_^2^ = 0.454, *p* < 0.001), followed by the cleaning methods (*η*_P_^2^ = 0.160, *p* < 0.001), the pressure during the air-abrasion (*η*_P_^2^ = 0.142, *p* < 0.001), the air-abrasion powder (*η*_P_^2^ = 0.099, *p* < 0.001), and aging (*η*_P_^2^ = 0.027, *p* < 0.001). SBS showed higher values than TBS (*p* = 0.001 – 0.033), except for initial measurements within specimens cleaned with BUT and pretreated with GP0.1 (*p* = 0.285). A positive correlation between SBS and TBS was found (*R*, 0.424, *p* < 0.001).

**Table 2:** Descriptive statistics (median, min/max) and 95% confidence intervals (CI) for SBS per cleaning method, pretreatment, and aging

	ISO			BUT			CEN		
	Median	Min/max	95% CI	Median	Min/max	95% CI	Median	Min/max	95% CI
Pretreatment	Initial								
AL0.1	35.5^bαII^	13.5/54.6	(26; 39)	34.4^bβi^	10.1/52.5	(35; 41)	46.0^aαII^	25.4/56.3	(37; 50)
GP0.1	36.0^aα^	16.1/50.3	(29; 41)	23.3^*bBγii^	1.6/34.9	(10; 28)	44.0^aαβ^	7.9/64.5	(28; 48)
AL0.4	43.3^bαII^	7.9/58.6	(30; 48)	37.1^bβiiII^	28.3/51.6	(34; 43)	49.2^aαII^	36.5/61.1	(45; 54)
GP0.4	38.4^bα^	8.0/49.5	(26; 43)	44.0^abAαi^	36.6/48.3	(40; 46)	45.2^aα^	38.0/62.0	(42; 52)
Control	34.3^aαII^	30.0/45.6	(31; 38)	38.2^aβII^	23.2/52.9	(33; 43)	33.1^aβII^	25.9/45.5	(31; 37)
Pretreatment	Artificial aging								
AL0.1	38.7^bBβI^	28.6/57.1	(35; 45)	45.7^bBβi^	10.1/62.6	(33; 51)	61.5^aαiI^	49.1/75.3	(57; 67)
GP0.1	36.2^*aβ^	22.4/66.2	(31; 47)	22.4^*aBγii^	3.1/53.0	(12; 35)	36.0^aBγii^	8.7/75.7	(26; 50)
AL0.4	55.0^aAαiI^	24.1/75.7	(44; 61)	56.8^aAαiI^	46.2/76.7	(52; 67)	67.0^*aαiI^	43.6/75.7	(56; 70)
GP0.4	41.3^bβii^	26.0/54.6	(37; 48)	42.3^bAβii^	15.0/55.0	(31; 46)	50.5^aAβii^	40.3/62.8	(46; 55)
Control	60.4^abαI^	38.4/75.5	(37; 48)	62.8^aαI^	52.7/75.7	(57; 67)	45.3^bβγI^	33.7/75.7	(41; 56)

*Not normally distributed. ^ab^Different lowercase letters present significant differences between the cleaning methods within one pressure, powder, and aging group. ^AB^Different uppercase letters present significant differences between the applied pressure within one powder, cleaning method, and aging group. ^αβγδ^Different letters present significant differences between pretreatments (1–5) within one cleaning method and aging group. ^i,ii^Different letters present significant differences between the applied powder within one pressure, cleaning method, and aging group. ^I,II^Different letters present significant differences between the aging regime within one pressure, powder, and cleaning group

**Table 3:** Descriptive statistics (median, min/max) and 95% confidence intervals (CI) for TBS per cleaning method, pretreatment, and aging

	ISO			BUT			CEN		
	Median	Min/max	95% CI	Median	Min/max	95% CI	Median	Min/max	95% CI
Pretreatment	Initial								
AL0.1	21.2^bBβI^	11.3/36.6	(16; 27)	16.2^bβ^	8.0/35.6	(11; 23)	30.6^aBβγ^	18.6/37.7	(25; 34)
GP0.1	19.5^abβ^	1.7/32.7	(12; 24)	13.9^bBγ^	2.6/27.4	(8; 20)	26.0^aδ^	6.2/39.1	(18; 31)
AL0.4	25.7^bAα^	17.3/48.0	(22; 35)	27.6^bα^	13.5/40.6	(21; 34)	38.0^aAα^	31.5/44.2	(34; 40)
GP0.4	20.7^*bαβ^	10.8/44.1	(16; 30)	18.6^bAβ^	8.5/38.8	(16; 30)	33.5^aαβ^	20.1/46.5	(27; 37)
Control	27.4^aαII^	17.6/42.2	(23; 33)	31.0^aαII^	21.5/41.5	(26; 34)	24.8^aγδII^	20.3/36.2	(22; 30)
Pretreatment	Artificial aging								
AL0.1	13.9^bBγII^	5.3/25.3	(10; 18)	17.6^bBβγ^	1.7/36.6	(10; 33)	26.7^aBβγ^	17.1/38.6	(24; 34)
GP0.1	18.8^aβγ^	10.2/34.7	(14; 25)	9.5^bBγ^	0.5/25.7	(4; 18)	20.4^aBγ^	9.8/41.8	(17; 29)
AL0.4	35.6^aAαi^	25.0/43.7	(30; 38)	35.3^aAαi^	23.8/41.0	(29; 37)	35.7^aAαi^	29.5/49.5	(32; 41)
GP0.4	17.9^bβii^	8.3/22.7	(14; 20)	15.9^bAβii^	10.3/28.6	(13; 22)	30.3^aAβii^	19.0/38.8	(25; 33)
Control	36.8^aαI^	23.3/41.9	(31; 39)	35.0^aαI^	29.0/41.0	(31; 37)	34.8^aαI^	26.3/44.2	(30; 38)

*Not normally distributed. ^ab^Different lowercase letters present significant differences between the cleaning methods within one pressure, powder, and aging group. ^AB^Different uppercase letters present significant differences between the applied pressure within one powder, cleaning method, and aging group. ^αβγδ^Different letters present significant differences between pretreatments (1–5) within one cleaning method and aging group. ^i,ii^Different letters present significant differences between the applied powder within one pressure, cleaning method, and aging group. ^I,II^Different letters present significant differences between the aging regime within one pressure, powder, and cleaning group

### SBS measurements

Regarding the cleaning methods, CEN led to higher values for groups pretreated with AL0.1 (*p* < 0.001–0.014), initially tested specimens pretreated with AL0.4 (*p* < 0.001–0.036), and the aged group pretreated with GP0.4 (*p* = 0.003–0.021). Specimens tested in the initial state and pretreated with GP0.4, cleaned with CEN, presented higher values compared to ISO (*p* = 0.006). ISO showed higher values for specimens tested in the initial state and pretreated with GP0.1 (*p* = 0.001) compared to BUT. The aged groups pretreated with GP0.1 or AL0.4 and the initially tested control group (*p* > 0.092) showed no difference in cleaning methods, whereas the aged control group showed higher values when cleaned with BUT compared to CEN (*p* = 0.002).

Regarding the pressure, 0.4 MPa led to higher values in artificially aged groups cleaned with ISO and pretreated with alumina (*p* = 0.006) or cleaned with CEN and pretreated with glass pearls (*p* = 0.029). In addition, groups cleaned with BUT and air-abraded at 0.4 MPa increased values in groups pretreated with glass pearls (*p* < 0.001–0.026) or pretreated with alumina after thermocycling (*p* = 0.004).

When comparing the different pretreatments 1 to 5, the highest values were observed in groups pretreated with AL0.4 (*p* < 0.001–0.019), except for BUT-cleaned initially tested specimens (*p* < 0.017), while pretreatment with GP0.1 showed the lowest values (*p* < 0.001–0.029).

Regarding the powder, pretreatment with AL0.4 showed higher values than GP0.4 (*p* < 0.001–0.010) in aged groups. Thermocycled groups cleaned with CEN or BUT and pretreated with AL0.1 presented higher values than GP0.1 (*p* < 0.001–0.012). BUT-cleaned initially tested specimens, pretreated with AL0.1, led to higher values than GP0.1 (*p* = 0.010), whereas pretreatment with GP0.4 (*p* = 0.019) led to higher values compared to AL0.4.

Regarding the aging regime, thermocycling increased SBS values (*p* < 0.001–0.029) when cleaned with ISO or CEN and pretreated with alumina. In addition, the control group (*p* < 0.001–0.002) and BUT-cleaned specimens pretreated with AL0.4 (*p* < 0.001) showed higher values after artificial aging.

### TBS measurements

Regarding the cleaning methods, CEN showed the highest values (*p* < 0.001–0.024) except for the control group (*p* > 0.220) and aged specimens pretreated with AL0.4 (*p* = 0.415). Cleaning with ISO compared to BUT led to higher values for thermocycled specimens pretreated with GP0.1 (*p* < 0.036). Significant differences between CEN and ISO were detected in groups pretreated with AL0.1 (*p* < 0.003) or GP0.4 (*p* < 0.011) and for initially tested specimens pretreated with AL0.4 (*p* = 0.017). Cleaning with CEN showed higher values compared to BUT for groups pretreated with AL0.1 (*p* < 0.001–0.004), GP0.1 (*p* = 0.003–0.007), or GP0.4 (*p* < 0.001–0.029) and for initially tested specimens pretreated with AL0.4 (*p* = 0.007).

Regarding the pressure level, 0.4 MPa increased TBS values in groups cleaned with ISO and pretreated with alumina (*p* < 0.001–0.034) and in artificially aged groups cleaned with BUT or CEN (*p* < 0.001–0.049). In addition, initially tested, 0.4 MPa led to higher values for BUT-cleaned specimens pretreated with glass pearls (*p* = 0.029) and centrifugated specimens pretreated with alumina (*p* = 0.002).

**Fig. 2:**
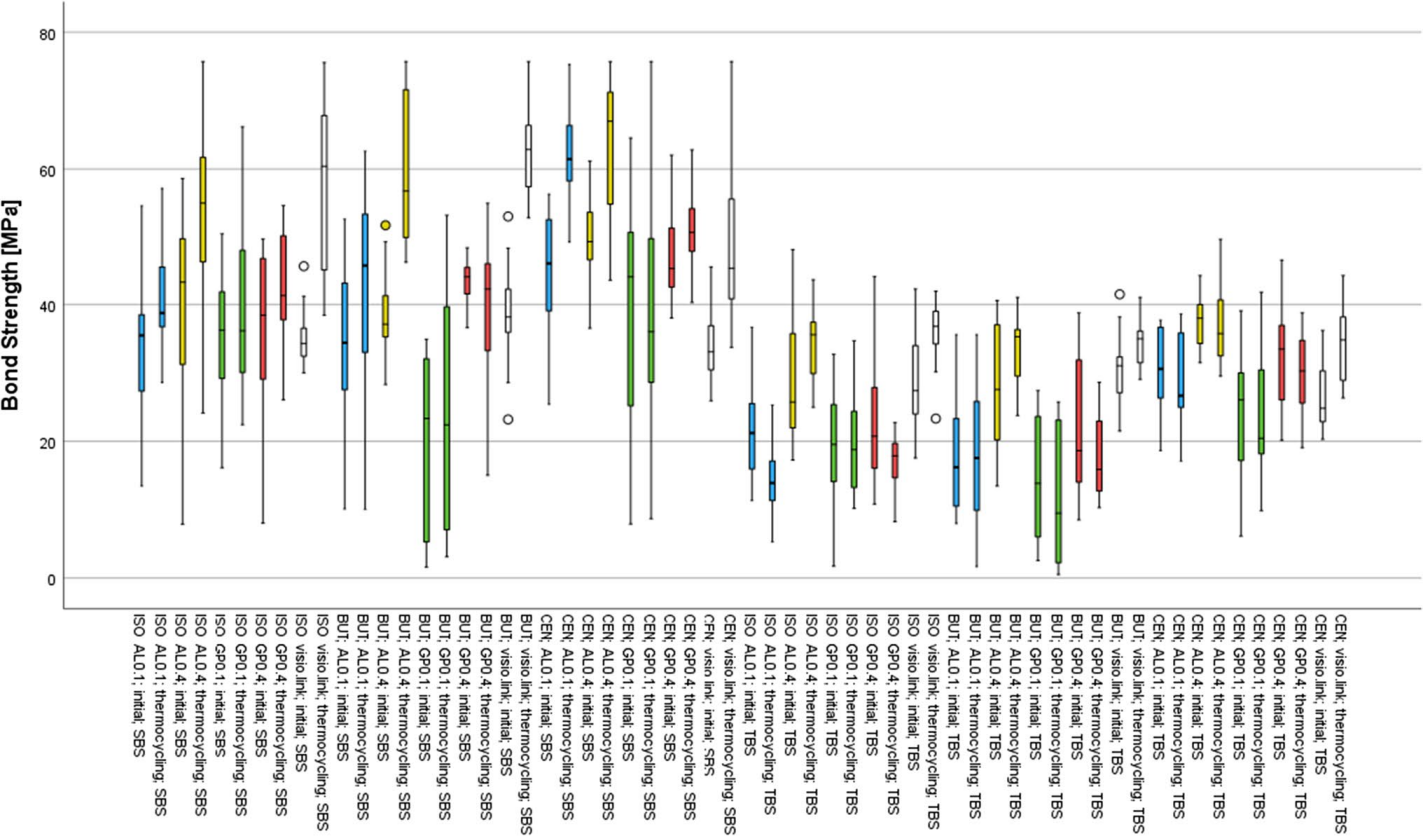
Bond strength values (MPa) of all tested groups

When comparing the different pretreatments 1 to 5, pretreatment with AL0.4 led to the highest values (*p* < 0.001–0.033), whereas GP0.1 showed the lowest values (*p* < 0.001–0.049) (Fig. 2).

Regarding the powder, after artificial aging, pretreatment with AL0.4 led to higher values than pretreatment with GP0.4 (*p* < 0.005). As for the aging regime, ISO-cleaned specimens pretreated with AL0.1 showed lower values (*p* = 0.010) after artificial aging. The control group presented higher values (*p* < 0.001–0.026) after 10,000 thermal cycles.

### SFE and Ra

The highest impact on Ra was exerted by pressure (Ra: *η*_p_^2^ = 0.610, *p* < 0.001) and followed by powder (Ra: *η*_p_^2^ = 0.382, *p* < 0.001). Air-abrasion with alumina (*p* < 0.001) or pressure at 0.4 MPa (*p* < 0.001) presented higher Ra values compared to specimens pretreated with glass pearls or 0.1 MPa pressure. The highest Ra values were observed by pretreatment with AL0.4 (Table 4).

**Table 4:** Descriptive statistics (median, min/max) and 95% confidence intervals (CI) of measured surface roughness Ra (µm) and SFE on particle-abraded specimens

	Ra			SFE		
Pretreatment	Median	Min/max	95% CI	Median	Min/max	95% CI
AL0.1	1.36^*b^	1.19/1.87	(1.23;1.53)	50.55^a^	45.60/56.10	(48.84;53.21)
GP0.1	1.31^b^	1.02/1.61	(1.21;1.51)	49.80^a^	46.70/54.50	(48.25;52.34)
AL0.4	2.23^a^	2.03/2.59	(2.12;2.38)	49.95^a^	46.30/50.90	(48.26;50.59)
GP0.4	1.52^b^	1.12/2.18	(1.34;1.83)	49.90^a^	43.90/50.80	(46.59;50.42)

*Not normally distributed. ^ab^Different letters present significant differences between pretreatment groups (1–4)

### Fracture types

**Fig. 3:**
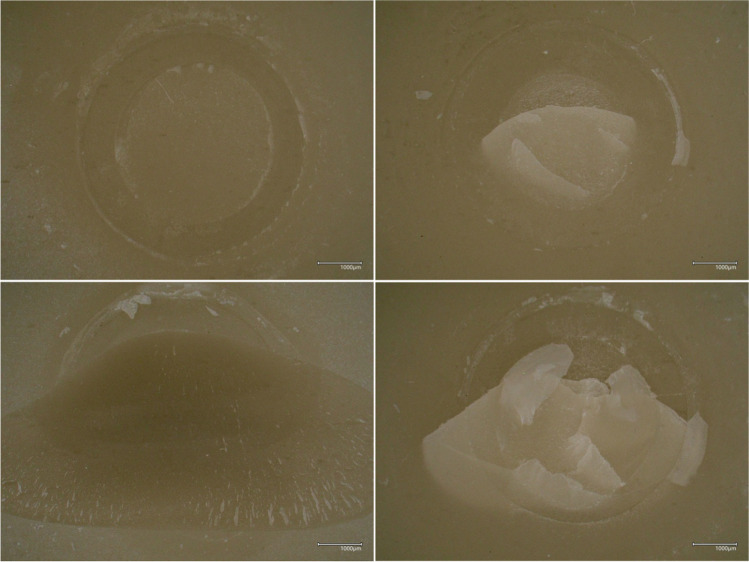
Digital microscope images of adhesive (top left), cohesive within the luting composite resin (top right), cohesive within the 3D-printed resin (bottom left), and mixed cohesive (bottom right) fractures

Digital microscopic images show the four fracture types evaluated (Fig. 3). 95% CI and percentage of investigated fracture types are summarized in Tables 5 and 6.

**Table 5:** Percentage of evaluated fracture types and 95% CI for SBS per cleaning method, pretreatment, and aging

Initial		% adhesive and 95% CI	% cohesive within luting composite and 95% CI	% cohesive within 3D resin and 95% CI	% mixed and 95% CI
Cleaning	Pretreatment				
ISO					
	AL0.1	13 (0; 41)	13 (0; 41)	73 (43, 93)*	0 (0; 22)
	GP0.1	13 (0; 41)	20 (3; 49)	67 (37; 89)*	0 (0; 22)
	AL0.4	7 (0; 32)	33 (10; 62)	47 (20; 74)	13 (0; 41)
	GP0.4	20 (3; 49)	13 (0; 41)	67 (37; 89)*	0 (0; 22)
	Control	0 (0; 22)	0 (0; 22)	100 (77; 101)*	0 (0; 22)
BUT					
	AL0.1	20 (3; 49)	13 (0; 41)	60 (31; 84)*	7 (0; 32)
	GP0.1	53 (25; 79)	0 (0; 22)	47 (20; 74)	0 (0; 22)
	AL0.4	0 (0; 22)	0 (0; 22)	73 (43; 93)*	27 (6;56)
	GP0.4	7 (0; 32)	13 (0; 41)	67 (37; 89)*	13 (0; 41)
	Control	0 (0; 22)	0 (0; 22)	100 (77; 101)*	0 (0; 22)
CEN					
	AL0.1	0 (0; 22)	27 (6; 56)	47 (20; 74)	27 (6; 56)
	GP0.1	47 (20; 74)	27 (6; 56)	20 (3; 49)	7 (0; 32)
	AL0.4	0 (0; 22)	33 (10; 62)	40 (15; 68)	27 (6;56)
	GP0.4	0 (0; 22)	0 (0; 22)	73 (43; 93)*	27 (6; 56)
	Control	0 (0; 22)	0 (0; 22)	100 (77; 101)*	0 (0; 22)
Artificial aging		% adhesive and 95% CI	% cohesive within luting composite and 95% CI	% cohesive within 3D resin and 95% CI	% mixed and 95% CI
Cleaning	Pretreatment				
ISO					
	AL0.1	0 (0; 22)	27 (6; 56)	73 (43; 93)*	0 (0; 22)
	GP0.1	0 (0; 22)	40 (15; 68)	60 (31; 84)	0 (0; 22)
	AL0.4	0 (0; 22)	13 (0; 41)	60 (31; 84)*	27 (6;56)
	GP0.4	0 (0; 22)	20 (3; 49)	80 (50; 96)*	0 (0; 22)
	Control	13 (0; 41)	13 (0; 41)	73 (43; 93)*	0 (0; 22)
BUT					
	AL0.1	20 (3; 49)	33 (10; 62)	47 (20; 74)	0 (0; 22)
	GP0.1	47 (20; 74)	13 (0; 41)	40 (15; 68)	0 (0; 22)
	AL0.4	0 (0; 22)	27 (6; 56)	53 (25; 79)	20 (3; 49)
	GP0.4	13 (0; 41)	20 (3; 49)	67 (37; 89)*	0 (0; 22)
	Control	0 (0; 22)	73 (43; 93)*	27 (6; 56)	0 (0; 22)
CEN					
	AL0.1	0 (0; 22)	33 (10; 62)	40 (15; 68)	27 (6; 56)
	GP0.1	27 (6; 56)	13 (0; 41)	60 (31; 84)*	0 (0; 22)
	AL0.4	0 (0; 22)	7 (0; 32)	40 (15; 68)	53 (25; 79)
	GP0.4	7 (0; 32)	40 (15; 68)	47 (20; 74)	7 (0; 32)
	Control	0 (0; 22)	40 (15; 68)	60 (31; 84)	0 (0; 22)

**Table 6:** Percentage of evaluated fracture types and 95% CI for TBS per cleaning method, pretreatment, and aging

Initial		% adhesive and 95% CI	% cohesive within luting composite and 95% CI	% cohesive within 3D resin and 95% CI	% mixed and 95% CI
Cleaning	Pretreatment				
ISO					
	AL0.1	40 (15; 68)	13 (0; 41)	40 (15; 68)	7 (0; 32)
	GP0.1	67 (37; 89) *	0 (0; 22)	33 (10; 62)	0 (0; 22)
	AL0.4	33 (10; 62)	13 (0; 41)	53 (25; 79)	0 (0; 22)
	GP0.4	40 (15; 68)	20 (3; 49)	40 (15; 68)	0 (0; 22)
	Control	0 (0; 22)	33 (10; 62)	67 (37; 89)*	0 (0; 22)
BUT					
	AL0.1	67 (37; 89) *	0 (0; 22)	33 (10; 62)	0 (0; 22)
	GP0.1	67 (37; 89) *	0 (0; 22)	33 (10; 62)	0 (0; 22)
	AL0.4	13 (0; 41)	20 (3; 49)	60 (31; 84)*	7 (0; 32)
	GP0.4	40 (15; 68)	20 (3; 49)	40 (15; 68)	0 (0; 22)
	Control	7 (0; 32)	27 (6; 56)	67 (37; 89)	0 (0; 22)
CEN					
	AL0.1	0 (0; 22)	20 (3; 49)	73 (43; 93)*	7 (0; 32)
	GP0.1	53 (25; 79)	20 (3; 49)	27 (6; 56)	0 (0; 22)
	AL0.4	0 (0; 22)	73 (43; 93)*	27 (6; 56)	7 (0; 32)
	GP0.4	7 (0; 32)	53 (25; 79)	40 (15; 68)	0 (0; 22)
	Control	0 (0; 22)	7 (0; 32)	93 (67; 100)*	0 (0; 22)
Artificial aging		% adhesive and 95% CI	% cohesive within luting composite and 95% CI	% cohesive within 3D resin and 95% CI	% mixed and 95% CI
Cleaning	Pretreatment				
ISO					
	AL0.1	73 (43; 93) *	0 (0; 22)	27 (6; 56)	0 (0; 22)
	GP0.1	40 (15; 68)	7 (0; 32)	53 (25; 79)	0 (0; 22)
	AL0.4	0 (0; 22)	40 (15; 68)	60 (31; 84)	0 (0; 22)
	GP0.4	47 (20; 74)	0 (0; 22)	53 (25; 79)	0 (0; 22)
	Control	13 (0; 41)	80 (50; 96)*	7 (0; 32)	0 (0; 22)
BUT					
	AL0.1	40 (15; 68)	13 (0; 41)	47 (20; 74)	0 (0; 22)
	GP0.1	73 (43; 93) *	0 (0; 22)	27 (6; 56)	0 (0; 22)
	AL0.4	0 (0; 22)	53 (25; 79)	47 (20; 74)	0 (0; 22)
	GP0.4	67 (37; 89) *	0 (0; 22)	33 (10; 62)	0 (0; 22)
	Control	0 (0; 22)	80 (50; 96)*	20 (3; 49)	0 (0; 22)
CEN					
	AL0.1	13 (0; 41)	13 (0; 41)	73 (43; 93)*	0 (0; 22)
	GP0.1	80 (50; 96) *	7 (0; 32)	13 (0; 41)	0 (0; 22)
	AL0.4	0 (0; 22)	40 (15; 68)	60 (31; 84)	0 (0; 22)
	GP0.4	0 (0; 22)	33 (10; 62)	67 (37; 89)*	0 (0; 22)
	Control	7 (0; 32)	67 (37; 89)*	27 (6; 56)	0 (0; 22)

For SBS, predominantly, cohesive fractures within the 3D-printed resin were observed (40–100%), except for groups cleaned with BUT or CEN and pretreated with GP0.1 where adhesive failures occurred (27–80%). Mostly mixed cohesive fractures were observed in centrifugated specimens pretreated with AL0.4. For TBS, groups showed predominantly cohesive fractures within the 3D-printed resin or cohesive fractures within the luting composite, except for groups pretreated with GP0.1, where adhesive fractures occurred (40–80%).

## Discussion

The range of applications of 3D printable resins in dental practice is excelling fast. However, the use of it as fixed dental protheses requires a permanently stable and durable adhesive bond via a luting composite resin. The bond between the luting composite resin and 3D-printed resin might depend on the post-processing procedures applied to the resin. A variety of cleaning methods and air-abrasion possibilities exists to be used with 3D-printed resin, all of which have not been researched so far in this context. With the present investigation, some of most promising combinations of these have been considered. Based on the results presented, the proposed hypothesis is rejected in all cases. Overall, among the cleaning methods, the highest SBS and TBS values were observed in combination with centrifugation. Using centrifugation, it was observed that a visibly thin layer of residual monomer covered the surface of the specimen, whereas the other two cleaning methods only left a blank surface and consequently a visibly more effective cleaning [18].

It is assumed that the higher concentration of residual monomers with unreacted double bonds on the resin surface, after post-polymerization in Otoflash G171 under nitrogen atmosphere, which improves the degree of conversion [19], could be exposed again by the mechanical pretreatment of the surface and have a positive influence on the bond between the 3D resin matrix and luting composite. This may be attributed to the unconverted double bonds following copolymerization. Preliminary measurements with cleaned substrates and without mechanical pretreatment already showed insufficient bond strengths initially, especially centrifuging, and non-pretreated substrates achieved bond strength values of zero. An investigation regarding the repair of 3D-printed resin substrates resulted in the recommendation to repair the printed substrates with temporary composite resin without mechanical pretreatment, but the substrates were not chemically or physically cleaned [15].

Studies done on splints created via AM have shown that conditioners containing methyl methacrylate (MMA) play a vital role in the durability of bond strengths [13]. Convincing results were also achieved with MMA in the bonding of CAD/CAM composite resin blocks [22]. Therefore, visio.link has been used as a positive control. visio.link consists of MMA, dimethacrylate, and pentaerythritol acrylate and thus generate an adhesive bond to the resin matrix. Contrary to the assumption that this use might have a positive impact on the bond strength, the results have shown that this control had a negative impact when combined with centrifugation as a cleaning method, whereas centrifugation in combination with any other air-abrasion pretreatment displayed the best results. A possible explanation could be that the 3D resin used contains 40% inorganic glass–ceramic fillers which may hinder the conditioning with MMA and therefore the bonding qualities. This assumption is reinforced via further investigations where resins containing less fillers in combination with the use of MMA conditioning displayed better bonding features [23]. In this investigation, the chemical cleaning methods in combination with MMA displayed higher bond strength. A possible explanation for this could be that these cleaning methods had a higher cleaning capacity than centrifugation since the chemical cleaning releases some of the fillers from the resin matrix [24], with which MMA can copolymerize, without fillers hindering. In particular when pretreated with glass pearls or low pressure, visio.link increases the bond strength, as higher pressure or alumina particles could expose the fillers again, which would impair the bond with visio.link. Regarding the fracture types, for conditioning with visio.link, mostly cohesive fractures within the 3D resin occurred, which indicates that the bond strength is higher than the flexural strength of the printed material. Pretreatment with alumina predominately produced better bonding features than a pretreatment with glass particles. When comparing the two pretreatment materials with regard to the shape, alumina, in unstructured shape of particles, displays a rougher surface than glass pearls, which are microspheres. This could lead to a better penetration of the resin which in turn could lead to a better bonding with the luting composite due to an improved interlocking [20, 23, 25]. This possible explanation is also supported by the different pressures used where higher pressures displayed better bonding features presumably due to the higher pressures resulting also in a deeper penetration of the resin surface. However, it is important to note that high pressure can damage the surface, resulting in the falling out of fillers and leading clinically to a possibly poorer fit of the restoration [26].

Thermocycling has established itself as suitable for simulating temperature changes in the oral cavity [27, 28]. In this study, half of the bond strength measurements were performed after thermal aging of 10,000 cycles between 5 and 55 °C. Ten thousand cycles are equivalent to about 1 year of use [27], but thermocycling is only an approximation for certain intraoral situations to simulate, e.g., hot food or ice cream. In this study, different results were found after aging on the SBS and TBS values. Increasing SBS values after thermal aging were reported in the previous investigation [29]. Lower values after artificial aging may be caused by mechanical stress in the bonding interface, caused by volumetric changes [30]. An increased bond strength can be explained by the upper temperature which can promote post-polymerization of the luting area. In addition, the absorption of water during thermal cycling causes 3D resin material to expand which may affect the anchorage of the luting composite resin.

In general, several bond strength measurement methods can be considered when evaluating adhesive properties. Among others, these can be macro-shear bond and macro-tensile bond strength tests [31] as well as micro-shear and micro-tensile tests. Micro-tests provide higher bond strength values than their equivalent macro-tests [32, 33] and work well for evaluating the dentin bond [34]. There are investigations questioning the clinical validity of bond strength in vitro tests [32, 35, 36]. However, due to their simplicity and being low technique-sensitive, the more commonly used macro-tests were applied [37, 38]. For macro-SBS and macro-TBS measurements, this study used the same specimen geometry and defined diameter of acrylic cylinders and thus an identical bonding area but different crosshead speeds. Within the limits suggested by ISO/TS 11,405, the crosshead speed does not seem to have any influence on the bond strength values [39]. Nevertheless, higher bond strength values were observed by SBS measurements than by TBS measurements; however, SBS (19–63 MPa) and TBS (12–38 MPa) showed similar tendencies. This was also reported earlier [40]. However, the measured values showed similar trends in the groups studied and can be compared with each other. It can be assumed that the differences of the qualitative test methods in the mean values are caused by the different types of force application. In the tensile test, the stresses at the bonding interface are much more homogeneous than those in the shear test, so that the maximum principal stress values are much closer to the nominal strength [39].

Another limitation of the study at hand is the fact that no a priori power analysis was performed to determine the sample size. The groups for post hoc power analysis were selected within isopropanol, as this is the most used cleaning procedure for 3D-printed objects and with the smallest dispersion. The post hoc power analysis comparing the results of aged specimens cleaned with ISO and pretreated at 0.1 MPa with glass pearls and the control group with VL within TBS measurements yielded a power of a two-sided two-sample *t* test of 100%, with a sample size of 15 specimens in each group, an observed effect of 16.64 MPa, and a pooled standard deviation of 7.34. However, it must be taken into account that for a few groups, especially the comparisons between the control group and the group pretreated with AL0.4 MPa, a smaller effect was observed, leading to a reduced power of the statistical analysis.

As new materials for dental restorations are launched every day, the optimal combination of substrates and bonding procedures is constantly evolving. In the present study, it was observed that the interaction between cleaning and pretreatment has an impact on the bond strength. Material combinations that passed the in vitro tests should be further investigated in long-term clinical trials.

## Conclusions

Within the limitations of the present in vitro study, the following conclusions could be drawn:

- The test methods, i.e., SBS and TBS, had the highest impact on bond strength values, whereby SBS overall resulted in higher values than TBS.
- Within the pretreatment groups, centrifuged specimens showed higher bond strength compared to the chemical cleaning methods investigated.
- The pressure has a greater influence on the bond strength than the type of air-abrasion powder. The pretreatment with GP0.1 showed the lowest bond strengths and the highest number of adhesive fractures.
- The pretreatment with AL0.4 seemed to have the highest bond strengths among the tested groups. Although SFE was not affected, air-abrasion with AL0.4 showed the highest Ra values.
- The control group presented equally high bond strengths as the pretreatment with AL0.4. The very high number of cohesive fractures in the 3D-printed material highlights the high bond strength.
- Artificial aging positively influenced the bond strength in almost all tested groups.

## Data Availability

All data generated or analysed during this study are included in this published article.
